# hnRNP A/B Proteins: An Encyclopedic Assessment of Their Roles in Homeostasis and Disease

**DOI:** 10.3390/biology10080712

**Published:** 2021-07-24

**Authors:** Patricia A. Thibault, Aravindhan Ganesan, Subha Kalyaanamoorthy, Joseph-Patrick W. E. Clarke, Hannah E. Salapa, Michael C. Levin

**Affiliations:** 1 Office of the Saskatchewan Multiple Sclerosis Clinical Research Chair, University of Saskatchewan, Saskatoon, SK S7K 0M7, Canada; patricia.thibault@usask.ca (P.A.T.); joseph.patrick.clarke@usask.ca (J.-P.W.E.C.); h.salapa@usask.ca (H.E.S.); 2 Department of Medicine, Neurology Division, University of Saskatchewan, Saskatoon, SK S7N 0X8, Canada; 3 ArGan’s Lab, School of Pharmacy, Faculty of Science, University of Waterloo, Waterloo, ON N2L 3G1, Canada; aravindhan.ganesan@uwaterloo.ca; 4 Department of Chemistry, Faculty of Science, University of Waterloo, Waterloo, ON N2L 3G1, Canada; subha.kalyaanamoorthy@uwaterloo.ca; 5 Department of Health Sciences, College of Medicine, University of Saskatchewan, Saskatoon, SK S7N 5E5, Canada; 6 Department of Anatomy, Physiology and Pharmacology, University of Saskatchewan, Saskatoon, SK S7N 5E5, Canada

**Keywords:** RNA binding protein, hnRNP A/B, RNA metabolism, splicing, RNA trafficking, RNA granules, RNA homeostasis, telomeres, neurodegenerative disease

## Abstract

**Simple Summary:**

The hnRNP A/B family of proteins (comprised of A1, A2/B1, A3, and A0) contributes to the regulation of the majority of cellular RNAs. Here, we provide a comprehensive overview of what is known of each protein’s functions, highlighting important differences between them. While there is extensive information about A1 and A2/B1, we found that even the basic functions of the A0 and A3 proteins have not been well-studied. We also noted that the regulation and tissue distribution of all four of the proteins and their different isoforms require further study. Finally, since these proteins together play such a central role in regulating the cell’s RNA, we call for careful comparative examination of these proteins to better define the precise boundaries of each protein’s role in cell function and disease.

**Abstract:**

The hnRNP A/B family of proteins is canonically central to cellular RNA metabolism, but due to their highly conserved nature, the functional differences between hnRNP A1, A2/B1, A0, and A3 are often overlooked. In this review, we explore and identify the shared and disparate homeostatic and disease-related functions of the hnRNP A/B family proteins, highlighting areas where the proteins have not been clearly differentiated. Herein, we provide a comprehensive assembly of the literature on these proteins. We find that there are critical gaps in our grasp of A/B proteins’ alternative splice isoforms, structures, regulation, and tissue and cell-type-specific functions, and propose that future mechanistic research integrating multiple A/B proteins will significantly improve our understanding of how this essential protein family contributes to cell homeostasis and disease.

## 1. A Brief History of the hnRNPs

Heterogeneous nuclear ribonucleoproteins, “hnRNPs”, were first defined as proteins associated with heterogeneous nuclear RNAs (hnRNAs), but not with chromatin, nucleolar RNAs, nor polyribosomal RNAs [1,2]. These “hnRNAs” were later understood to be pre-mRNAs and spliced mRNAs that had not yet left the nucleus. Early exploration of hnRNPs was largely chemical and biochemical in nature, with initial studies isolating purified cellular nuclei, and employing sucrose and cesium chloride gradients to isolate protein:RNA complexes that sedimented at approximately 40S. RNase treatments and cutting-edge technologies like UV-crosslinking, two-dimensional gel electrophoresis, and Edman protein sequencing were then employed to define the chemical characteristics and amino acid sequences of the proteins of interest [3,4,5,6,7,8,9,10]. Speculation about protein function was limited since individual hnRNPs’ preference for DNA or RNA binding, and any sequence specificity, was not immediately apparent [2,10,11,12]. Instead, in electron micrographs, hnRNPs appeared to bundle nuclear RNAs much the same way histones bundle chromosomal DNA [13]. Since these protein complexes appeared to be exclusively found in the nuclear fraction of cells [14,15], and proteins with similar molecular weights and isoelectric points (pI) were occasionally associated with chromatin, they were hypothesized to bundle and store newly-transcribed mRNAs until the mRNAs were needed in the cytoplasm [2,9,12].

With increasingly sophisticated biochemical tools, including the advent of monoclonal antibodies [10,16,17,18], hnRNPs were better defined as approximately twenty-five different nuclear proteins (hnRNP A through U) with varying affinity for hnRNAs [7,19]. Some hnRNPs were separately identified and characterized in other contexts, and are now more commonly known by other names: Auf1 (hnRNP D), PCBP1 & 2 (Poly(RC) binding proteins 1 & 2; hnRNP E1 & E2), RBMX (RNA binding motif protein, X-linked; hnRNP G), PTB-1 (Polypyrimidine tract binding protein 1; hnRNP I), FUS (fused-in-sarcoma; hnRNP P2), SYNCRIP (Synaptotagmin binding cytoplasmic RNA interacting protein; hnRNP Q). However, a core subset of six hnRNPs (hnRNP A1, A2, B1, B2, C1, and C2) were found to be highly expressed, and consistently tightly associated with hnRNAs [9,20]. Of these, hnRNP A1 and A2 were most abundant, representing approximately 60% of the hnRNP mass in HeLa cells [9]. All six species were found to have affinity for nucleic acids, from single-stranded DNA (ssDNA) to poly-adenylated RNAs and purified hnRNAs. hnRNP C proteins demonstrated the highest affinity for RNAs, and an acidic pI of 5.9 [9]. In contrast, the hnRNP A and B proteins were distinguished by their highly basic pIs ranging from 8.2 to 9.6, with the additional unusual characteristics of high glycine content and enriched asymmetrical di-methylated arginine content [7,9,10]. Thus, without knowledge of their domain architecture, enzymatic activities, or function, the hnRNP A/B protein family (herein collectively referred to as A/B proteins) was defined.

**Figure 1:**
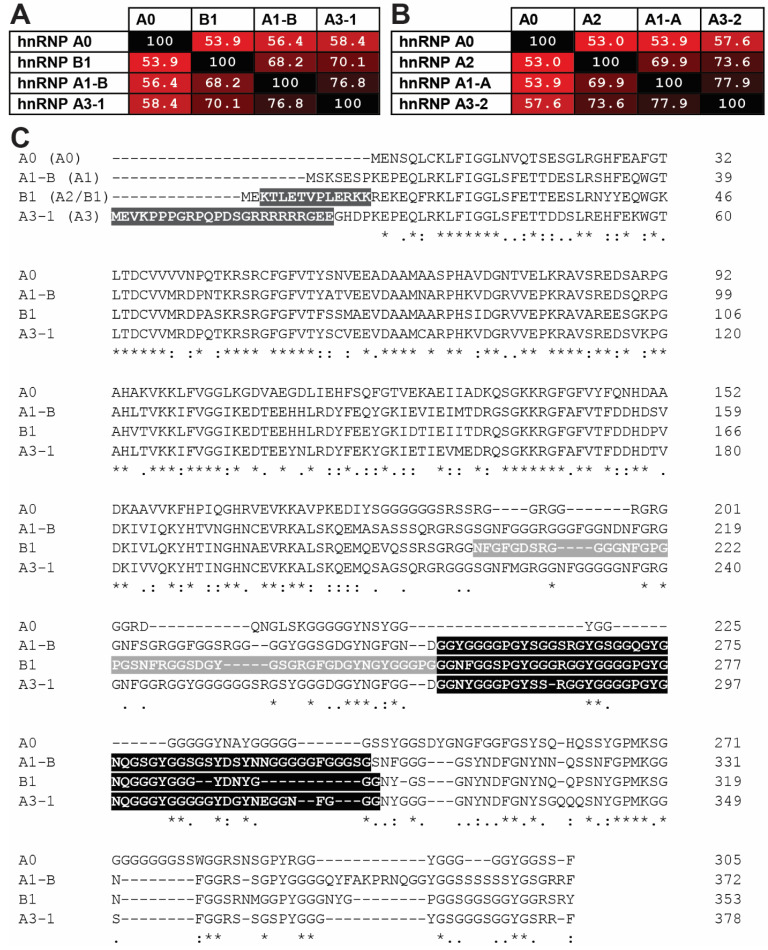
Human hnRNP A/B proteins are closely related and share similar alternative exonic structures. Protein sequences from the full-length isoform for each hnRNP A/B protein family member were aligned using ClustalW [26]. (**A**) Percent identity matrix for the full-length isoforms for each hnRNP A/B protein family member: A0 (NCBI Protein accession number NP_006796.1); A1-B (NP_112420.1); B1 (NP_112533.1); A3-1 (NP_001317178.1). Boxes are shaded red to indicate degree of sequence identity. (**B**) Percent identity matrix for the most common isoforms for each hnRNP A/B protein family member, coloured as in (**A**). A0 (NP_006796.1); A1-A (NP_002127.1); A2 (NP_002128.1); A3-2 (NP_001317176.1). (**C**) Sequence alignment for the full-length isoforms of each A/B family protein, from (A). Black and grey highlights indicate exons excluded in alternative isoforms as depicted in Figure 3 (e.g., amino acids 251–302 in A1). Note that the first exon of hnRNP A3 is replaced with an exon encoding just “ME” in isoforms A3-2 and A3-4.

**Figure 2:**
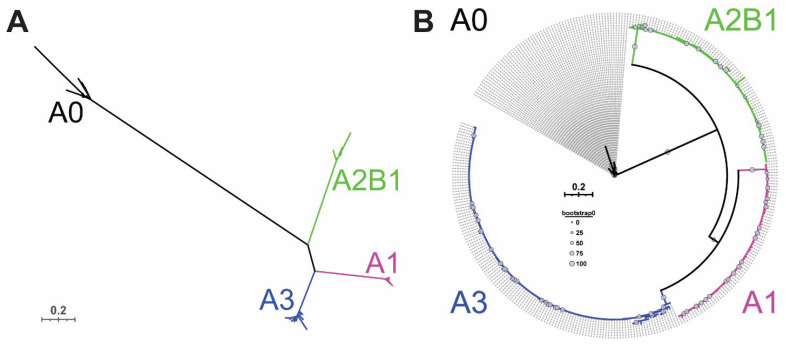
Updated analysis of mammalian hnRNP A/B protein sequences demonstrates their evolutionary relatedness. Sequences were drawn from the NCBI Orthologs database for each hnRNP A/B family protein, and manually depleted of Low-Quality protein sequences. Different isoforms of each protein within a species were retained. Sequences were then aligned using a Mafft multiple sequence alignment program [27], identical sequences were manually removed, and ambiguous sites were masked using Alistat [28], before generating maximum likelihood trees with VT + F + R4 model in the IQTree program [29]. Original inputs: A0, *n* = 140; A1, *n* = 259; A2/B1, *n* = 428; A3, *n* = 352. (**A**) Unrooted phylogram of mammalian hnRNP A/B family proteins, coloured by protein of origin. Scale bar indicates 0.2 amino acid changes per site. (**B**) Circular tree (polar plot) representation of mammalian hnRNP A/B family proteins with A0 as outgroup. Each dotted line indicates a unique sequence; duplicate sequences have been removed from the plot. Grey circles indicate bootstrap confidence values.

This review will focus upon the hnRNP A/B protein family members, specifically A1, A2/B1, A0, and A3, outlining their cellular function and highlighting their roles in disease pathogenesis. In particular, while the hnRNP A/B proteins share significant amino acid sequence identity (Figure 1) and are all involved in multiple aspects of cellular RNA metabolism, there is clear evolutionary evidence (Figure 2), supported by experimental data, that each protein fills a distinct role in cellular homeostasis. For example, all four proteins share a high N-terminal amino acid identity (Figure 1C), but their C-terminal domains are more divergent in amino acid sequence and protein:protein interactors, facilitating their below-described distinct roles in the cell.

To our knowledge, this is the first review to comprehensively address hnRNPs A0 and A3. It should be noted that: (1) a gene and protein product named *HNRNPA1L2* (A1-like 2) that shares significant protein sequence similarity to the known hnRNP A/B proteins [21] is identified in NCBI, Uniprot, and Ensembl’s databases [22,23,24], but lacks any functional data, and (2) the confusingly-named *HNRNPAB* (protein: hnRNP AB or CBF-A) and *CIRBP* (protein: cold-inducible RNA binding protein, hnRNP A18) are evolutionarily distinct from the hnRNP A/B family proteins [25]; thus, these proteins will not be discussed further in this review.

## 2. hnRNP A/B Proteins: An Overview

**Figure 3:**
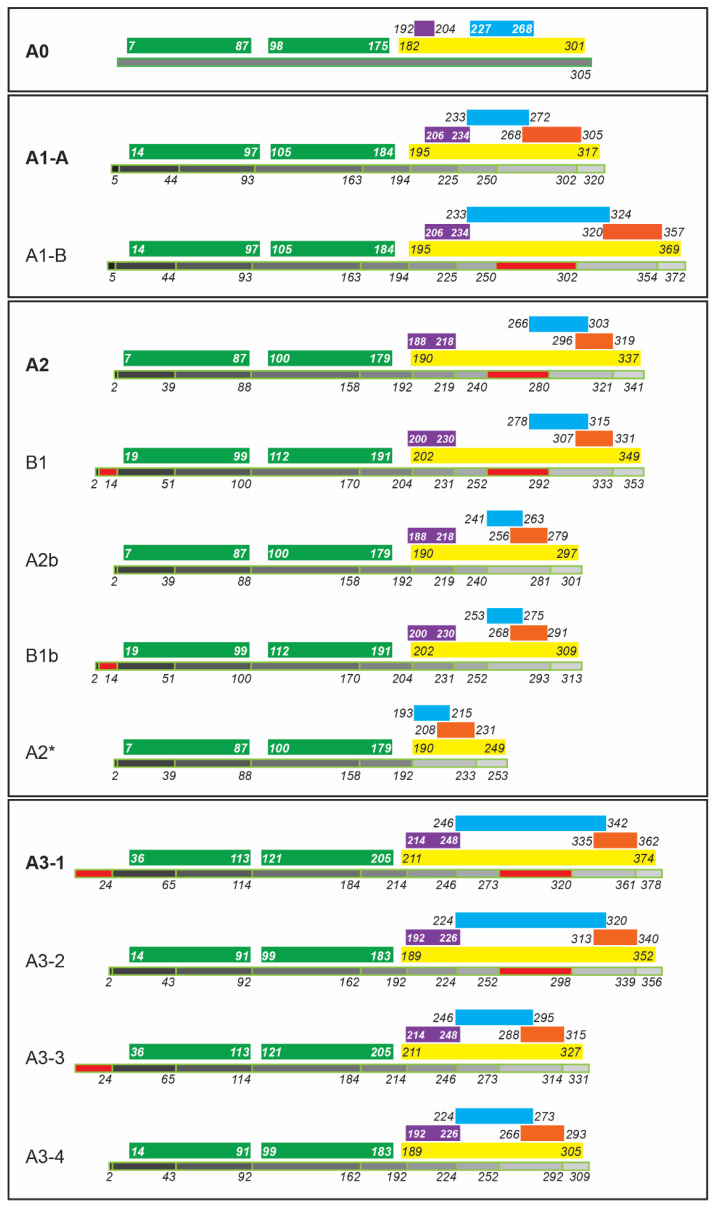
Domain architecture of hnRNP A/B family proteins and their known isoforms. All regions are to-scale. The total protein for each isoform is in black-and grey, with each shade corresponding to an exon. Red exons are altered in splice variants: for example, note that the A1-A isoform lacks amino acids 251–302 found in the longer A1-B isoform. Green denotes RNA recognition motifs RRM1 (left) and RRM2 (right), and yellow denotes the glycine-rich region, or low-complexity domain (LCD). Within the glycine-rich region, purple denotes RGG (arginine-glycine-glycine) motif-containing region, blue denotes the core prion-like domain (PrLD), and orange denotes the M9 nuclear localization sequence. Note that A0 does not bear an annotated M9 sequence motif. A0 and A3 RGG regions were inferred by the authors, while the A0 and A3 PrLDs were defined using PLAAC prediction algorithms [43].

**Figure 4:**
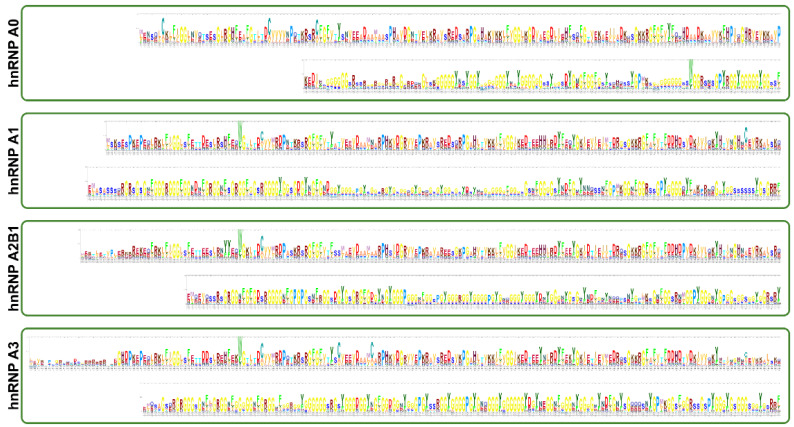
Sequence logos for the Hidden Markov Model (HMM) profile of the longest isoforms of each hnRNP A/B protein, generated using the Skylign program [48] through the HMMER implementation available within Skylign.

Structurally, the hnRNP A/B proteins consist of two N-terminal RNA recognition motifs (RRMs) and a C-terminal glycine-rich domain that is often referred to by its structural nature as a low-complexity domain, or LCD. The glycine-rich LCD further encompasses an RGG motif (arginine-glycine-glycine) region, an M9 nuclear localization sequence, and an intrinsically-disordered core prion-like domain (PrLD) (Figure 3 and Figure 4) [19,30]. A1, A2/B1, and A3 share a common exon/intron architecture (Figure 3) [31], supporting the proposal that A2/B1 and A3 originate from gene duplications of A1 [32], with A0 being a later product of erroneous retrotransposition of an A2/B1 mRNA [25]. This is supported by antigenic analysis [33] and our phylogenetic analysis (Figure 2). Since it was derived from reverse transcription of mRNA, the *HNRNPA0* gene lacks an intron/exon structure and encodes a single isoform, A0 (Table 1) [33]. *HNRNPA1* encodes two isoforms of hnRNP A1, A1-A and A1-B (Table 1, Figure 3). A1-A (often simply referred to as A1) is the dominantly-expressed isoform and lacks the expanded PrLD of A1-B (amino acids 251–302, Figure 1C and Figure 3) [34,35]. *HNRNPA2B1* encodes two major isoforms, with A2 generally being significantly more abundant than B1 [36]; unlike A1, both A2 and B1 contain the exon encoding an expanded PrLD. *HNRNPA2B1* also encodes three more-recently identified and less-abundant isoforms, A2b, B1b, and A2* (Table 1, Figure 3) [37,38], which all lack the expanded PrLD exon. Finally, *HNRNPA3* encodes at least four isoforms (A3-1, -2, -3, and -4; Table 1, Figure 3), but the relative abundance of these isoforms appears to vary among animal species, and also likely varies within different tissues [39,40]. *HNRNPA3* and its isoforms are now known to be the origins of the hnRNP proteins previously labeled as hnRNP B2, mBx, and FBRNP (fetal brain ribonucleoprotein) [16,37,39,41,42]. Despite the large body of literature on hnRNP A1 and A2/B1, and the burgeoning research on hnRNP A0 and A3, there is a clear gap in the literature regarding differing functions of the various A/B proteins’ isoforms.

hnRNP A/B proteins and their varying isoforms are remarkably well-conserved. For example, the amino acid sequence distance between all known mammalian A1 proteins is vanishingly small despite an input of >250 sequences (Figure 2), and A2/B1 and A3 show a similar tight conservation within each protein (Figure 2 and Figure 4). The strongest homology is found in the RRMs (Figure 1). The clear separation between the proteins, as well as the sequence conservation of each protein and its isoforms [25,31], supports the proposition that each hnRNP A/B family protein is essential, but non-redundant. Together, hnRNP A/B proteins impact the metabolism of RNA polymerase II-derived RNAs [44] (micro RNA (miRNA), long non-coding RNA (lncRNA), and mRNA) from biogenesis to degradation.

Classically, hnRNP A/B proteins do not demonstrate enzymatic activities such as nuclease or polymerase activities. Instead, they can be envisioned as molecular intermediaries, functioning to promote, protect, and traffic RNAs within the cellular environment in response to the cell’s ever-changing needs. Under homeostatic conditions, hnRNP A/B proteins are primarily localized to the nucleus where they function in positive and negative regulation of transcription initiation, promotion of alternative splicing, and facilitate the translocation of mRNAs from nucleus to cytoplasm. However, Friend et al. [45] noted that A3 and A2/B1 demonstrate distinct intranuclear localization from A1. A3 and A2/B1 are more diffuse and perinucleolar at all stages of the cell cycle, while A1 is enriched at the nuclear membrane [45]. The precise effect of this difference is not known, but it is an early observation that indicates that each A/B protein carries out functions distinct from the otherwise closely-related family members. In response to various stimuli (e.g., osmotic stress, cytokine signaling [46,47]), hnRNP A/B proteins traffic and store mRNAs in varying types of nuclear and cytoplasmic RNA:protein granules, modulate RNA stability, and regulate cap-dependent and internal ribosomal entry site (IRES)-mediated translation. However, careful comparison of the A/B proteins with each other (below) shows that within these broad functions, the A/B proteins each have distinct roles.

By being so deeply integrated into all levels of cellular RNA metabolism, hnRNP A/B proteins impact an enormous range of disease processes, including metabolic diseases, neurodegenerative diseases, developmental disorders, viral infections, and cancers. They also have the potential to provide a wealth of information about maintenance of RNA homeostasis in different cells, tissues, and organisms.

## 3. hnRNP A1 and A2/B1

hnRNPs A1 and A2/B1 are the most abundant proteins of the 40S ribonucleoprotein complex [9]. They are best characterized for their roles in regulating cellular RNA homeostasis and have been shown to modulate essentially every aspect of mRNA metabolism including transcription, splicing, nucleocytoplasmic transport, translation, storage, and stability. These functions are critical for maintaining the viability of both cell and organism, and so most pathology associated with dysfunction of these proteins is defined by alterations to mRNA metabolism. In addition, A1 and A2/B1 are involved in telomere maintenance and combine this with regulation of mRNA to modulate organismal development and cellular differentiation. Not unexpectedly, breakdown in this regulation is associated with oncogenesis. Altered RNA metabolism and dysfunction of A1 and A2/B1 are also associated with neurodegenerative diseases. In addition, A1 and A2/B1 have long been recognized as autoimmune targets, and recent work provides evidence that autoantibodies are not merely a by-product of autoimmune disease, but instead can pathologically impact A1 and A2/B1 function. For a specific and comprehensive review of A1 biology, we recommend Clarke et al. [51], and for the same of A2/B1, we recommend Liu & Shi [52].

A1 and A2/B1 are expressed in most tissues, but are found highly expressed in neurons of the central nervous system (CNS) [53]. Both A1 and A2/B1 have two distinct major isoforms (Table 1, Figure 3). The primary A1 isoform is approximately 20-fold more abundant than the A1-B alternative isoform, which is the product of the introduction of an additional exon that expands both its glycine-rich LCD and PrLD. Relatively little is understood about what purpose these different abundances serve, but one group has demonstrated that the expanded PrLD of A1-B also increases the pathogenic aggregative propensity of the protein [54]. Of the *HNRNPA2B1*-encoded proteins, A2 is the primary (shorter) isoform, while B1 has an extra 12 amino acids inserted at the N-terminus of the protein, preceding the RRMs. The relative abundance of A2 and B1 varies drastically in different tissues, while other splice variants are much less-well characterized [37,38]. With some exceptions that will be noted, most research has been carried out using or evaluating the primary isoform of each protein, or not distinguishing between them.

### 3.1. DNA Binding: Telomere Regulation, Transcription

Although hnRNPs are characterized by their interactions with RNA, both A1 and A2/B1 also have known DNA binding activity that serves multiple cellular purposes. Both proteins interact with telomeric DNA and aid in both its protection and regulation of telomerase activity [21,55]. A1 and A2/B1 can bind to telomeres’ G-rich ssDNA overhang when it forms a G-quadruplex structure [50,56,57,58,59], which prevents its detection by the cell’s DNA damage response [60,61]. In vitro, A1 has also been shown to promote telomerase activity [58], and in culture systems, depletion of either or both A1 and A2/B1 results in altered telomere elongation and/or shorter telomeres over cell replication [60], while overexpression increases telomere length [62]. Uniquely, Wang et al. [49] also found that a low-abundance shorter isoform of A2/B1, named A2*, (Table 1, Figure 3) has a more specific affinity for telomeric DNA than the major A2/B1 isoforms, binds telomeric RNA (hTR), immunoprecipitates with telomerase, and unwinds the telomeric structure to enhance telomerase processivity. Surprisingly, to our knowledge, the role of A1 and A2/B1 in altered telomere maintenance has yet to be linked to oncogenesis (see, for example, Shishkin et al. [21] or Roy et al. [63]). Importantly, most of the work describing the interactions of A1 and A2/B1 with telomeres has been done in the context of transformed cell lines, and so the magnitude and direction of effects vary in healthy tissues and organisms [64], or under different stimuli [65].

As direct transcriptional activators/repressors, A1 and A2/B1 bind to the promoter regions of a specific gene, as is seen with A1 binding to promoter sequences for repression of vitamin D receptor transcription [66] or cell-cycle-regulated repression of human thymidine kinase transcription [67]. In these instances, both A1 and A2/B1 specifically recognize a G-quadruplex structure, like that in telomeric DNA, in many of the promoter regions they regulate [68,69,70,71]. A1 and A2/B1 are found to be overexpressed in a number of cancers [63,72,73,74,75]. This is characterized to result in an aberrant up-regulation of transcriptional targets like *TRA2B* (encoding Transformer 2β) in colon cancer [68] or *KRAS* (encoding Kirsten rat sarcoma viral oncogene homolog) in pancreatic cancer [69,71]. Both of these oncogenes are implicated in the initiation of a number of different cancers [76,77], and so these findings broadly connect both A1 and A2/B1 regulation of transcription to oncogenesis. These and other transcriptional targets of A1 and A2/B1 in cancer are reviewed in refs [21,63].

In addition to G-quadruplex-facilitated binding, A2/B1 seems to be guided to particular promoter regions by noncoding RNAs. Small RNAs guide A2/B1 to the *CDKN1A* (cyclin-dependent kinase inhibitor 1a; p21^Waf1/Cip1^) promoter to induce its transcription and regulate cell cycle progression [78], while long non-coding RNAs direct A2/B1 to specific promoters to both down- [79,80] and up-regulate [81] transcription. A1 and A2/B1 are both known to interact with a small nuclear RNA (7SK) to facilitate release of P-TEFb (positive transcription elongation factor b) and promote transcription [82,83]. A2/B1 also interacts with the transcription factors CREB (cyclic-AMP responsive element binding protein), NF-κB (nuclear factor kappa B), and C/EBPδ (CCAAT/enhancer-binding protein delta) to promote their activation and induce a transcriptional response to mitochondrial stress [84,85]. A recent publication from the same group suggests that A2/B1 has lysine acetyl-transferase activity, and acts as a histone acetyltransferase to regulate mitochondrial stress-induced gene expression [86]. They also propose that A2/B1 histone modifying activity regulates telomerase length under mitochondrial stress [65]. This would be the first classical enzymatic activity attributed to hnRNP A/B family proteins. Thus, while both A1 and A2/B1 have transcriptional modulation functions, to our current knowledge, A2/B1 uses a much wider range of mechanisms to do so.

### 3.2. RNA Splicing

hnRNP proteins A1, A2, and B1 were first characterized as major components of the heterogeneous nuclear 40S RNA:protein complex, which is now understood to be pre-mRNAs and mature mRNAs complexed with splicing and transport factors [87]. All hnRNP A/B proteins are found in the spliceosome E complex [88]. Under homeostatic conditions, A1 and A2/B1 bind to splicing enhancer sequences for introns and alternative exons to exclude them from the mature mRNA, antagonizing alternative splice factor proteins [30,89,90,91]. However, recent high-throughput work suggests that A2/B1 is biased towards binding activated exons [92] and 3’UTRs after splicing is complete [93]. Stimuli like hormone or growth factor signaling, acute stress induction, or innate immune activation and cytokine signaling, all result in reduced A1 RNA binding, permitting incorporation of alternative exons and retention of introns in the mRNA [51]. These stimuli likely also modulate A2/B1, although this has not been thoroughly explored. Reduced RNA binding by A1 and A2/B1 results in either production of alternatively-spliced protein isoforms, or nonsense-mediated decay of mRNA with retained intronic sequences [46,91,94,95,96,97,98]. Reduced RNA binding to facilitate alternative splicing appears to be mediated by post-translational modifications of A1 such as phosphorylation or ubiquitination [51], but recent research also suggests that non-coding RNAs play a role in modulating the presence of A1 and A2/B1 at specific splicing suppressor and enhancer regions [99,100,101]. Given that non-coding RNAs direct A2/B1 in the context of transcription, it is likely that future research will find a role for them in regulating A2/B1 in splicing.

Alternative splicing functions as a short-term response to a stimulus like cellular stress; when the stress is removed, A1 and A2/B1 resume suppressing alternative splicing. However, a long-term stimulus such as aberrant growth factor signaling or innate immune signaling can result in the accumulation of alternatively-spliced proteins, and ultimately to negative outcomes like oncogenesis and metastasis [68,97,102,103] or sensitivity to programmed cell death [104,105]. In the instance of the genetic disorder spinal muscular atrophy (SMA), A1-mediated suppression of splicing prevents complementation of defective *SMN1* (survival motor neuron 1) by an alternative splice isoform of *SMN2* [106,107,108]; a therapy targeting this splicing mechanism was recently approved, but viral gene therapy providing intact *SMN1* has superseded this approach [109]. A2/B1 regulation of splicing is also implicated in Alzheimer’s disease (AD), where Berson et al. [110] found that both A1 and A2/B1 were depleted in entorhinal cortex samples from AD patients, with evidence of increased alternative RNA splicing. Loss of acetylcholine and resultant loss of cholinergic neurons is a hallmark of AD. Genetic mouse models that increased cholinergic signaling also increased A2/B1 protein levels [110,111], while in a range of genetic mouse models for loss of cholinergic signaling, A2/B1 protein levels were severely reduced in the affected neurons, mRNA splicing profiles were altered [112], and the mice developed hallmarks of AD neurodegeneration [110]. A1 depletion drove alternative splicing of the *APP* gene encoding amyloid-β (Aβ), involved in pathogenic plaque formation in AD, thereby increasing abundance of its more pathogenic splice variant [113], indicating multiple contributions of A1 and A2/B1 to alternative splicing in AD. Of note, among the many targets of A1 and A2/B1 splicing regulation are their own mRNAs, *HNRNPA1* and *HNRNPA2B1* [92,114,115], which provides an avenue to disrupt runaway dysregulation [116], but itself represents a potential major point of failure for aberrant responses to stimuli.

### 3.3. RNA Trafficking

After splicing, A1 and A2/B1-containing protein:mRNA complexes traffic to the cytoplasm [117], facilitated by the M9 nuclear localization and nuclear export sequence present in all major isoforms of A1 and A2/B1 (Figure 3) [118]. Although the details of nuclear export are not yet well-characterized, A1 does interact with NXF1 (nuclear export factor 1), an adaptor that facilitates mRNA transition through the nuclear pore complex [119]. Upon delivery of their mRNA cargo to sites of translation or storage, A1 and A2/B1 return to the nucleus by a specific interaction with the nuclear import factor Transportin-1 (TNPO1, Karyopherinβ-2) [120,121] that requires RanGTP hydrolysis to release the hnRNP proteins back into the nucleus to repeat the cycle [122,123]. This process is heavily regulated by post-translational modifications, where either *O*-GlcNAcylation [124] or SUMOylation [119] may mark cytoplasmic RNA-depleted A1 for return to the nucleus, while methylation, phosphorylation, acetylation, and PARylation may all prevent such a return [51]. Various stimuli, from growth factor and cytokine signaling to osmotic stressors and heat shock, drive these post-translational modifications and modulate A1 trafficking for rapid and short-term regulation of mRNA availability [51,125]. It currently appears that A2/B1 localization can be modulated by methylation [125,126,127], but future work will be required to clarify what regulates this for each isoform. Since post-translational modifications are relatively-poorly explored in A2/B1, proteomics approaches [128] may provide insight into whether analogous modifications to A1 also regulate A2/B1 localization and activities.

Once within the cytoplasm, A2/B1 in particular has multiple key roles in trafficking RNAs. A2-mediated transport of mRNAs is best described in neurons, and thoroughly reviewed by Smith et al. [129]. A2/B1 binds to a short consensus sequence known as an A2-response element (A2RE) [130] in select mRNAs in either the nucleus or the cytoplasm, and catalyzes assembly of RNP trafficking granules [131,132]. Han et al. [133] have found that the isoform A2b (Figure 3, Table 1) is predominant in the neuronal processes (axons and dendrites) and cytoplasm under homeostatic conditions, and is critical for assembly of RNA trafficking granules in rats. It is not yet clear whether this is the case in human nervous tissue, and Han et al. note the absence of A2b expression in other, non-neuronal cell lines [133]. These granules traffic along microtubules to their destinations within the cell (e.g., distal dendritic sites [134]) where the mRNAs are stored until needed for a translational response [129,132,135,136]. SUMOylated A2/B1 is also heavily involved in miRNA transport in the cytoplasm [137], partnering with Annexin-2 (ANXA2) to traffic select miRNAs to exosomes for secretion to neighboring cells and tissues—and in at least one instance, sequestering a miRNA from exosomal release [138]. Interestingly, A2/B1 protein is found in miRNA-containing exosomes secreted in the central nervous system [139], but not in liver or kidney-derived cell lines [140]; the significance of this is unknown. Finally, A1 has only recently been identified to contribute to exosomal packaging of miRNAs, and only in the context of cancer [141,142], so its homeostatic role has yet to be determined.

### 3.4. miRNA Regulation

miRNAs are processed in a mechanism analogous to splicing: capped transcripts encoding the primary miRNA sequence (pri-miRNA) are produced by RNA polymerase II, and then the precursor miRNA (pre-miRNA) hairpin is excised from this transcript by the nuclear enzymes Drosha and DGCR-8 (DiGeorge Syndrome critical region gene 8). The pre-miRNA is then shuttled from nucleus to cytoplasm via Exportin-V for final processing by Dicer, and loaded into the RNA-induced silencing complex for effector activity [143]. A1 is a known positive and negative regulator of this process. It binds to the hairpin loop in the pri-miRNA encoding miR-18a using both RRM1 and RRM2, weakens the double-stranded hairpin stem, and facilitates Drosha-mediated processing into pre-miR-18a [144,145,146]—all without affecting processing of the other miRNAs on the same transcript [147]. A1 was also found to bind to the pri-miRNA of let-7a [146], and sterically hinder KSRP (KH-type splicing regulatory protein) access to the hairpin, resulting in a reduced abundance of mature let-7a miRNA [148]. In contrast, A2/B1 is best characterized to regulate miRNA trafficking described above. However, A2/B1 overexpression was found to change the miRNA profile of epithelial adenocarcinoma cells [149]. A2/B1 was also found to bind 6-methyladenosine (m^6^A) modifications [150], which are enriched on pri-miRNA transcripts, and Alarcón et al. [150] and Chen et al. [151] suggest that A2/B1 recruits DGCR8 to promote pri-miRNA processing.

The combination of miRNAs and splicing are mechanistically implicated in the link between A1, A2/B1, and oncogenesis. Many miRNAs implicated in cancer development have themselves been identified to regulate A1 and A2/B1 in a series of associative studies, although these tend not to produce a direct connection to oncogenesis [152,153,154,155,156]. However, a series of recent work has tied A1, A2/B1, miR-Let-7a, miR-206, the oncogenic long non-coding RNA LINC01234, CRY2 (cryptochrome 2), PKM2 (pyruvate kinase M2, an oncogenic splice variant of PKM1), and c-Myc in a complicated web of reciprocal modulation that, when dysregulated, induces an oncogenic transcriptional and splicing profile in a number of cancers [151,157,158,159,160].

### 3.5. mRNA Translation, Stability, and Granules

A1 and A2/B1 have a regulatory role to play in translation through their RNA binding activities. Both proteins are known to bind to AU-rich elements (AREs, not to be confused with A2-response elements, A2REs, mentioned above) [161,162,163,164,165,166], destabilizing sequences that are generally found in the 3′ UTR of mRNAs for cytokines and other rapid-response genes [167,168]. This binding is used to either repress their translation [46], or promote mRNA degradation [169]. A1 and A2/B1 are also known as internal ribosomal entry site (IRES)-transactivating factors (ITAF) and bind to the RNA structures of cellular IRESes to facilitate and regulate cap-independent translation of mRNAs [51,170,171]. Under stress or microbial invasion, cells will halt cap-dependent translation to preserve resources and deny intracellular pathogens access to cellular machinery, while essential and responsive mRNAs will contain IRES structures that recruit alternative factors for translation initiation in the absence of cap-binding proteins. A1 responds to stressors and other signals by binding to IRES sequences and facilitating their translocation to the cytoplasm for cap-independent translation. In both instances, post-translational modifications are the means by which the cell rapidly induces changes in A1 facilitation of translation [51]. Many viruses also contain IRES structures in order to bypass cellular restrictions, and will hijack A1 for their own benefit; this was recently reviewed by Kaur and Lal [172], although it is important to note that they use a non-standard naming convention for A1 isoforms. A1-mediated altered translation has been implicated in cellular proliferation and cancer, where growth factor receptors and oncogenes like *FGF-2* (fibroblast growth factor 2), *MYC* (c-Myc), *IQGAP1* (IQ motif containing GTPase activating protein 1), and *CYLD* (cyclin D1) are aberrantly translated [119,166,173,174,175]. Although the regulation of A2/B1 release of translational suppression or ITAF promotion is not yet known, mRNAs are trafficked by A2/B1 to neuronal dendrites in non-translating RNP granules, and its subsequent phosphorylation is what permits mRNAs to be translated [176]. While this suggests a common mechanism with A1, in a lone study, A2/B1 acted as an anti-proliferative in breast cancer cells by suppressing translation of a proliferation protein, Profilin 2 [177]. Thus, it is clear that we cannot generalize from A1 to presume A2/B1 function.

Finally, A1 and A2/B1 are well-characterized to play a role in the biology of stress granules (SGs). SGs are a distinct form of RNP complex that uses liquid–liquid phase separation (LLPS) to establish membrane-less structures that are nonetheless distinct from the cytoplasmic milieu [178]. This is achieved by interactions between the PrLDs of proteins like G3BP (Ras GTPase-activating protein-binding protein 1), FUS, TDP-43 (TAR DNA binding protein 43), and TIA1 (T-cell intracellular antigen-1) [178,179]. The primary function of SGs is to act as storage sites for mRNAs and other RNAs whose translation has been halted in response to stressors [178,180]. Once stress stimulus has been removed, SGs disassemble and release stored mRNAs to resume translation [178,181]. A1 and A2/B1 can promote SG assembly and/or associate with SGs due to their PrLDs, which also mediate interaction with other SG proteins [181,182,183,184,185,186,187].

As with the other aspects of A1 and A2/B1 responses to stress, SGs themselves can be dysregulated. It is most apparent in neurodegenerative diseases like amyotrophic lateral sclerosis (ALS), frontotemporal dementia (FTD), and multiple sclerosis (MS), where disease-associated genetic and somatic mutations in the PrLD of A1 and A2/B1 result in their increased cytoplasmic localization, exacerbated and expedited response to stress stimuli, and delayed or inhibited granule disassembly [51,52,93,187,188,189,190]. This and other hnRNP-related neurodegenerative disease mechanisms have been recently reviewed by Bampton et al. [184] and by Clarke et al. [51]. Insoluble protein aggregates occur when granules are no longer able to disassemble [178,191]. Biological mechanisms for protein recycling are less effective due to aggregate size and insolubility, and so aggregates accumulate in pathologic fibrils that can lead to cell death and in neurons, neurodegeneration [192,193,194]. For diseases like ALS, A1 and A2/B1-containing pathogenic aggregates can be seeded by accumulation of other PrLD-containing RNA binding proteins (RBPs) including TDP-43, FUS, and TIA1 [178,182,183,195]. A1 nuclear depletion, cytoplasmic mislocalization, and co-localization with SG markers is found in neurons of MS brains, an autoimmune disease with a significant neurodegenerative component [186,196,197]. Further, in a mouse model of MS, degree of A1 mislocalization and SGs are associated with disease severity [198,199]. We hypothesize that this is either initiated or exacerbated by the autoimmune inflammatory signaling in MS [200,201].

RBP aggregates have additional pathogenic effects outside of the aggregates themselves. Cytoplasmic sequestration of A1, A2/B1, and other RBPs can cause their nuclear depletion and therefore have significant downstream effects on the cellular transcriptome and proteome [93,192,196]. For example, nuclear depletion of TDP-43, a hallmark of ALS, results in alternative splicing of *HNRNPA1* itself and production of A1-B, the A1 isoform with an expanded PrLD of increased aggregative propensity, creating a positive feedback loop of aggregation and nuclear depletion of PrLD-containing proteins [54]. Other studies show that increased phospho-TDP-43-positive inclusion bodies in neurons correlated with nuclear depletion of A1 [202], and increases in A2/B1 mRNA [203]. As the above descriptions of mRNA metabolism indicate, total mRNA levels often do not correspond to protein levels, and it is possible that either depletion of A2/B1 protein (similar to A1) results in increased mRNA transcription, or that alternative splicing of the *HNRPNA2B1* mRNA occurs as it does for *HNRNPA1* and affects protein levels differently from mRNA levels—or that both mechanisms are involved.

Autoimmunity has an interesting role to play in A1 and A2/B1 mislocalization and aggregation as well. hnRNP A/B family proteins have long been recognized as autoantibody targets in a subset of autoimmune diseases like rheumatoid arthritis (RA), systemic lupus erythematosus (SLE), mixed connective tissue disease, scleroderma, Sjorgren’s syndrome, and MS [41,204,205,206,207,208,209,210,211,212]. Originally proposed as simple biomarkers of disease [206,213,214,215,216], these antibodies are now hypothesized to play a pathogenic role. In animal models for both MS and RA, antibodies to A1 and A2/B1, respectively, exacerbate disease. Autoantibodies, including those targeting A1 and A2/B1, have been shown to be internalized within cells of the central nervous system and interact with their intracellular protein targets [217,218,219]. MS autoantibodies to A1 in neurons primarily target its M9 nuclear localization sequence [210], localizing with SGs and trapping A1 in the cytoplasm, and ultimately preventing it from carrying out essential nuclear functions and resulting in neuronal cell death. In SLE, autoantibodies against double-stranded DNA cross-react to A2/B1 and promote its methylation, which is thought to exacerbate its cytoplasmic accumulation [217]. Additionally, other autoantibodies in SLE targeting brain cytoplasmic RNAs compete with A2/B1 for RNA binding sites and disrupt A2/B1 trafficking of RNAs to synapto-dendritic sites in neurons [134,218]. These findings indicate that autoantibodies have pathogenic potential by disrupting the normal functions of hnRNP A/B proteins.

## 4. hnRNP A0

Evolutionarily, hnRNP A0 is likely a “new” member of the hnRNP A/B family. Encoded in a single exon, A0 is hypothesized to have resulted from mistaken reverse transcription, followed by chromosomal integration, of an hnRNP A2-like mRNA [25]. It has 53–56% amino acid identity to the major A2/B1 isoforms, and 58% identity with A3 (Figure 1B). Within mammals, A0 is considerably more variable than the hnRNP core proteins A1 and A2/B1 (Figure 2). A0 displays the same basic structural characteristics of the other A/B proteins (Figure 3 and Figure 4), and was first found present on hnRNP-associated RNA sequences and in immunoprecipitated hnRNP complexes in much lower abundance than the other hnRNP proteins [33]. It has since also been detected by mass spectrometry in the spliceosome [88] and has a similar heavily di-methylated signal at R291 to that found in the other A/B proteins [220]. Aside from a single phosphorylation site described below, other post-translational modifications of A0 have not been discovered. However, A0 migrates at a heavier-than-expected molecular weight on two-dimensional gel electrophoresis, suggesting other post-translational modifications. It is likely that A0 was made “heavier” and/or changed apparent pI by post-translational modifications, but the degree of change (30.5 kDa to 38 or 39 kDa) suggests that this was mediated by more than a single modification [105,221]. Outside of specific pathogeneses described below, A0 was identified along with other hnRNPs (including other A/B proteins) to influence alternative splicing of proteins with intrinsically disordered regions [222]. The hnRNP A/B PrLDs are intrinsically-disordered regions, and alternative splicing of A1 and A2/B1 regulates the expansion of the PrLD. Such recursive self-regulation appears to be a theme among the hnRNP proteins. Since A0 has not been as thoroughly investigated as A1 and A2/B1, here we will synthesize the known literature in the context of broader biological questions.

### 4.1. Cellular Proliferation and Cancer

The vast majority of the literature has explored A0 function in the context of cancer, cell cycle progression, and apoptosis. Associative studies have implicated mutations in the upstream transcriptional regulatory region for A0 (c.-110G > C) in increased risk for colorectal cancer [223] and familial early-onset cancer [224], and identified hypermethylation of the gene as a risk factor for poor clear cell renal carcinoma prognosis and metastasis [225]. On a regulatory level, activation of E2F1 (E2a binding factor-transcription factor 1 [226]), a transcription factor that regulates cell cycle progression, or knockdown of SRBD1 (S1 RNA binding domain 1), an RNA-binding protein overexpressed in non-small cell lung carcinoma, both reduced A0 protein abundance, and resulted in increased sensitization to apoptosis, slowed proliferation in tissue culture, and reduced tumour sizes in animal models [227,228]. 

Reinhardt et al. [229] found that MAPKAP kinase 2 (MK2), an enzyme found to phosphorylate A0 at Serine 84 [47], did so in response to genotoxic stress (DNA damage). This phosphorylation event resulted in A0 binding and stabilizing the mRNA for Gadd45α (growth arrest and DNA-damage-inducible 45 alpha), which ultimately paused cells at the G2/M phase and prevented cell cycle progression. Later research further implicated the MK2-A0 axis as a linchpin regulator of cell cycle progression, a cytoplasmic backup when the master regulator p53 fails [230]. In addition to stabilizing *GADD45a* mRNA, Cannell et al. [230] also demonstrated that in the absence of p53, MK2-phosphorylated A0 stabilizes *CDKN1B* mRNA (encoding cyclin-dependent kinase inhibitor 1B, or p27^Kip1^) in response to doxorubicin treatment, which stalls the cells at the upstream G1/S checkpoint. In the absence of functional p53, this successful checkpoint stalling prevents DNA damage-induced apoptosis and confers resistance to chemotherapeutic agents. Both Cannell et al. [230] and Konishi et al. [231] showed that knockdown of A0 increased the susceptibility of lung, colon, gastric, pancreatic, and esophageal cancer cell lines to anti-proliferative chemotherapy. A0 knockdown also reduced tumour volume in transplanted mouse tumours [231]. Interestingly, growth of non-transformed cell lines was not impacted by A0 knockdown [231], likely because of intact p53 regulation that suppresses A0 abundance [230].

Zhang et al. [232] have demonstrated A0 RNA binding to regulate apoptosis. A0 interacts with A2/B1 and ELAVL1 (Embryonic-lethal, abnormal vision, Drosophila-like 1, aka HuR, another RBP) to bind *ANXA2R* (Annexin-2 receptor) mRNA, promote translation from a decoy upstream open reading frame, and prevent translation of the full Annexin-2 receptor protein. Knockdown of A0 resulted in full-length protein production and annexin-independent apoptosis [232]. Combining A0 knockdown and RNAseq with RIPseq (RNA immunoprecipitation and sequencing) in a colon cancer cell line, Konishi et al. [231] identified 26 mRNAs explicitly stabilized by A0 binding, and found that knockdown of three of these mRNAs themselves induced cell cycle arrest and apoptosis. MK2-mediated phosphorylation of A0 in non-transformed cells resulted in destabilization of these same mRNAs, and stabilization of an entirely different set of 36 mRNAs (identified by RIPseq), demonstrating that A0 has context-dependent distinct RNA binding preferences [231]. Collectively, this data paints a clear picture of A0 as a key regulator of cell cycle progression, where dysregulation in the context of other cancer-related mutations results in aberrant resistance to chemotherapeutic agents.

### 4.2. Intracellular Immune Signaling

Outside of explicit cancer and apoptotic signaling, other groups have connected A0 to intracellular immune signaling. In the context of myeloid cells and their precursors, Young et al. [233] found that reduction of A0 protein levels by either patient-derived genetic deletion or siRNA knockdown dysregulated hematopoietic differentiation and altered cytokine mRNA stability. The genetic deletion was associated with myeloid neoplasms, but they were unable to provide a direct oncogenic mechanism, and a number of other genes were also lost. Using biotinylated 3′ UTRs, a number of groups have identified A0 binding to the 3′ UTR of *PTGS2* (encoding COX2, cyclooxygenase 2), *CSF2* (GM-CSF, granulocyte-macrophage colony stimulating factor), *CXCL8* (IL-8, interleukin 8), *CXCL2* (MIP-2, macrophage inflammatory protein 2), and *TNF* (TNFα, tumour necrosis factor α) mRNAs [46,47,234,235], all of which contain AREs in their 3′ UTRs. Of note, these mRNAs were not identified in the combined RNAseq/RIPseq approach by Konishi et al. [231]. *CXCL8* and *CXCL2* mRNA binding was specifically induced by innate immune stimulation with lipopolysaccharide (LPS) [47,235], while Rousseau et al. [47] demonstrated that MK2 phosphorylation of A0 also promoted interaction with *TNF, CXCL2,* and *PTGS2* mRNAs. A0 has also been found to bind to regulatory elements in the pig coronavirus Transmissible Gastroenteritis virus RNA genome [236], and the human Hepatitis C virus RNA genome [237,238], but the consequences of these interactions are unknown. Finally, A0 has been implicated in binding un-methylated CpG motifs in exogenous oligodeoxyribonucleotides (CpG ODNs) in murine and salmon cells, translocating from nucleus to cytoplasm upon stimulation and thus acting as a sensor of bacterial-derived DNA in the same way toll-like receptors do [239,240]. Thus, here the nucleotide binding capacity of A0 appears to be employed as a tool for rapid cellular response to microbial invasion.

### 4.3. Neurobiology

Dammer et al. [241] sought to examine the nuclear protein content of human brain by nuclear isolation and sorting for neuronal *versus* glial populations, and found that A0 is enriched in the nuclei of neurons over non-neuronal cells like astrocytes or microglia. This is concomitant with other A/B family proteins, which are also particularly enriched in neurons of the CNS [53]. A0 has also been found to be enriched in the prefrontal cortex of schizophrenic patient brains [242], while a reduction of A0 levels in peripheral blood monocytes was predictive of aging-related cognitive decline in a longitudinal study [243]. In a separate study, a coding single-nucleotide polymorphism (chr5: 137088945, C/T > S/G) in A0 is associated with suicide attempts in a Utah-based study [244]. The only mechanistic neuronal study was carried out in a mouse model for Tau-proteinopathy in Alzheimer’s disease, where A0 was found to interact with and co-localize with cytoplasmic SG markers and diffuse phospho-Tau protein during the early stages of disease. Interestingly, this was also associated with increased A0 insolubility, a hallmark of pathogenic aggregation [245]. A0 accumulation in the cytoplasm was also observed in response to DNA damage-induced MK2 phosphorylation [230], and CpG ODN treatment in salmon [239]. Taken together, this suggests that like the other hnRNP A/B proteins, A0 translocates to the cytoplasm in response to stressful stimuli. However, further research is needed to identify the regulation and potential pathogenic outcomes of this localization.

## 5. hnRNP A3

Like the other hnRNP A/B family members, the amino acid sequence (Figure 1 and Figure 2) and splicing profile of *HNRNPA3* is highly conserved between humans and rodents [246]. The expression levels of these isoforms varies between organisms and between tissues in ways that have yet to be characterized, but are likely to be informative about A3 biological functions [39,40]. *HNRNPA3* encodes at least four isoforms (Table 1, Figure 3) that together account for multiple proteins previously identified as mBx, hnRNP B2, and FBRNP [16,41,247,248]. Two A3 isoforms were found to be the origins of the elusive “hnRNP B2” protein mentioned previously, which was detectable on SDS-PAGE and 2D gel electrophoresis, but whose mRNA and gene proved challenging to identify [37]. Definition of all the mammalian A3 isoforms as a unique set of proteins was facilitated by characterization of autoantibodies found in patients with rheumatoid autoimmune diseases [41]. As mentioned above, autoantibodies to other hnRNP A/B proteins have been found in rheumatoid autoimmune diseases. However, there were patients in these and other cohorts who produced autoantibodies with a different hnRNP binding profile that instead included the elusive B2 protein [16,41,211,215,247,249]. Using positive selection methods, Siapka et al. [41] were able to extract “B2”-binding antibodies that cross-reacted to some isoforms of the new A3 proteins, and using these antibodies to immunoprecipitate proteins for mass spectrometry, found that B2 and A3 proteins had identical peptides that were not present in the other hnRNP A/B proteins. This work connects A3 to autoimmune disease mechanisms shared by other hnRNP A/B proteins, where patients develop autoantibody responses to these essential and constitutively-expressed RBPs [207].

Regulation of A3 is only partially characterized. Under cellular stress and transformation-promoting conditions, A3 was phosphorylated at one or more of Serines 355, 356, or 358 [250]. This is analogous to osmotic stress-induced phosphorylation of A1 near its C-terminus [251] (compare within Figure 4), but unlike A1, the consequences of this phosphorylation are unknown. Additionally, when treated with Granzyme A (an apoptosis inducing secretory molecule), nuclear A3 abundance decreased more than tenfold, but the mechanism and specific outcome of A3 protein reduction was not explored [105]. Under homeostatic conditions, A3 is SUMOylated in the isoleucine-lysine-glutamate-aspartate (IKED) motif of RRM1 [252], is N-terminally acetylated (Methionine 1), and most of the arginines in its glycine-rich LCD are di-methylated, similarly to A1, its closest homolog (Figure 1 and Figure 2) [126]. This methylation may be carried out by protein arginine methyltransferase 3 (PRMT3) [253]. The precise drivers or purposes of these modifications is yet unknown.

Like A0, there is a body of literature surrounding A3 that is essentially associative. These findings will be excluded unless they provide context or confirmation of mechanistic studies. Also like A0, A3 has been characterized largely in the context of other biological questions, and so here we will synthesize the literature in similar contexts. Overall, A3 follows the broad theme of the hnRNP A/B family involvement in regulation of cell cycle progression and senescence, RNA metabolism, and neurobiology, with some suggestions as to regulation of response to innate immune stimuli.

### 5.1. Cellular Proliferation and Senescence

Much like A0, A3 has a clear regulatory role in cell cycle progression, cellular proliferation, and senescence, with overexpression promoting cell cycle progression and proliferation, and downregulation being a hallmark of cellular differentiation and senescence. Its regulation in these contexts is less-thoroughly explored. A3 overabundance has been associated with cancers (e.g., [254]), and treatment of rat glioma cells with glucocorticosteroids to revert their transformation phenotype also reduced A3 nuclear abundance [255]. During spermatid maturation, A3 expression was precipitously reduced prior to transition from round to elongated spermatid [256]. Li et al. [227] found that induction of the apoptotic-sensitizing transcription factor E2F1 reduced A3 protein abundance along with the other hnRNP A/B family members, although the mechanism of this regulation is unexplored. In these examples, a direct connection between stimulus and A3 protein reduction was not examined, but Comegna et al. [257] meticulously described how a senescence-promoting miRNA, miR-494, downregulates A3 protein abundance as a means of driving fibroblast senescence. Other miRNA studies were not as rigorous, and so they are difficult to interpret. Overall, it is clear that cells have mechanisms to down-regulate A3 nuclear or total abundance to suppress proliferation and promote differentiation.

The means by which A3 modulates cell cycle progression are still not well-understood. A3 has been found to interact with Sox-2, one of the Yamanaka factors required to induce stem cell pluripotency, but the outcome of this interaction was not explored [258]. The interaction between A3 and EGFR (epidermal growth factor receptor) is somewhat better-characterized: EGFR relocalizes to intracellular locations in response to stimuli like radiation and ligand binding, and when nuclear, can act as a transcriptional activator of oncoproteins like cMyc, Cyclin D1, and COX2 [259]. A3 and EGFR are both increased in expression in human lung cancers [254,259]. In cell culture studies, A3 interacted with nuclear EGFR [260], while its depletion also reduced EGFR nuclear localization [259]. This suggests that abundant A3 promotes EGFR nuclear accumulation and oncogene transcription. A3 was also implicated in a differentiation-induced alternative splicing mechanism to downregulate expression of the differentiation activator protein GRHL3 (Grainyhead Like Transcription Factor 3) in epidermal cells [261]. Other mechanisms by which A3 regulates proliferation have yet to be identified.

Telomere length is both a marker and driver of senescence [262]. Multiple groups have shown that A3 binds specifically to the G-rich strand of telomeric repeat DNA and affects cellular senescence [126,263,264,265,266]. Huang et al. [264,265] found that this binding required only RRM1, although RRM2 further improved binding affinity. The G-rich strand is the scaffold for a “cap”-like structure that prevents the cell from recognizing telomeres as damaged DNA, and since Tanaka et al. [263] noted that A3 binding prevents nuclease degradation of telomeres, while Travina et al. [267] recently found that A3 interacts with TRF2 (TTAGG repeat factor 2), they propose that A3 serves as a component of that protective cap. This straightforward picture of the role of A3 in telomere biology is complicated by two seemingly-conflicting findings: (1) in vitro, A3 suppresses both the telomerase reaction and DNA synthesis on the telomeric DNA [263], implicating it in negative regulation of telomeres, but (2) knockdown of A3 in cell lines with already-shorter telomeres resulted in additional telomere shortening over time [265], implicating it in telomere length maintenance. Studies of the effect of A3 in different cellular contexts (transformed versus immortalized cells) and types, and a better understanding of A3 abundance and localization regulation will likely enable us to resolve these findings.

### 5.2. RNA Metabolism

Like its better-characterized counterparts A1 and A2/B1, A3 is involved in multiple stages of RNA metabolism, from splicing, through nuclear-cytoplasmic shuttling, to translational regulation. Like the other A/B proteins, A3 is found in the spliceosome E complex and has been associated with alternative splicing [88,222]. A3 was also found to interact with a brain-specific small nucleolar RNA (snoRNA) called *MBII-52* (also known as *SNORD115*, and *HBII-52* in human tissues) [268]. This snoRNA is the only one currently known to regulate alternative splicing, and appears to do so using sequence complementarity to recruit splicing regulators to the serotonin receptor 2C (*5HT2C*) mRNA [269], among others [270], and thus it is likely that A3 contributes to this unusual means of RNA-mediated RNA regulation. A3 was also implicated in another form of alternative splicing regulation known as intronic polyadenylation (IpA). Polyadenylation signals result in cleavage at the signal, effectively deleting the remainder of the mRNA [271], so the retention of an intron containing a cryptic polyadenylation site results in either truncated proteins or nonsense mRNAs and prevents production of full-length protein. Chen et al. [261] sought to define the mechanism of change in IpA in keratinocyte differentiation, and found A3 interacts with cleavage and polyadenylation specificity factor complex proteins. Compared to other interactors, knockdown of A3 had the greatest effect on IpA-regulated gene *GRHL3*, and altered the cellular IpA RNA profile to a differentiation-like signature [261].

A3 is known to traffic mRNAs from nucleus to cytoplasm [39,272], and most of its characterized substrates contain an A2RE [126], including the mRNA for myelin basic protein, which is abundant in the CNS [272]. Once cytoplasmic, A3 was shown to interact with “turnover and translation regulatory RBPs” [273] like ELAVL1 [274], and ILF3 (interleukin enhancer binding factor 3), the long isoform of NF90 (nuclear factor of T-cells 90kDa) [275]. Katahira et al. [276] found that A3 interacts with nuclear export factor 7 (NXF7) in cytoplasmic RNA granules and in granules in the processes of cultured neurons. However, A3 and NXF7 do not interact in polysomes, and the cytoplasmic granules were characterized as P-bodies (processing bodies) that function to degrade RNAs, leading the authors to suggest that NXF7 and A3 interact to regulate faulty RNAs in their first “pioneering” round of translation [276]. Overall, our understanding of the ways in which A3 modulates mRNA processing is in its infancy, but researchers have identified interesting candidate pathways and genes to explore.

### 5.3. Intracellular Immunity

Like other hnRNP A/B proteins, A3 binds 3′ UTRs of mRNAs containing AREs like *COX2* and *TNF*, particularly when cells are stimulated with a toll-like receptor ligand like LPS [47,277]. It also binds regulatory elements in Hepatitis C virus and in the coronavirus MHV (mouse hepatitis virus) [238,278]. The consequences of these interactions, however, have yet to be defined. Knockdown of ISG15 (interferon-stimulated gene 15) expression, a complicated small protein that is both a post-translational modification (ISGylation) and an intracellular signaling molecule [279], resulted in reduction in A3 mRNA and protein levels. This was assessed in liver cells to test replication of Hepatitis C virus, but the authors used a viral construct where translation regulation was disconnected from the regulatory region bound by A3, and so the mechanism and outcome of ISG15-mediated A3 reduction was not determined [280]. Finally, A3 was found to interact with the nuclear RNA editing protein APOBEC3B (apolipoprotein B mRNA editing enzyme, catalytic polypeptide-like 3B) in an RNA-independent manner, but the effects of this interaction were not examined [281]. Thus, there are limited reports implicating A3 in regulation of intracellular immunity, but there is still insufficient information to assemble a mechanistic framework.

### 5.4. Neurobiology

When Ma et al. [39] first characterized A3, they observed its expression in rodent testes, spleen, lung, and brain. In particular, A3 puncta were found in neurites from cultured hippocampal mouse neurons, but these puncta were distinct from A2-containing RNPs, indicating a role in trafficking of specific transcripts in neurons [39]. Quite recently, Lopes et al. [282] studied squid A3, showing that it forms a homodimer in RNA-rich regions in synaptosomes of squid neurons, and binds specific mRNAs at the synaptic terminal. Thus, the authors concluded that A3 plays a role in RNP formation for site-specific RNA processing [282]. A3 was also recently shown to play a critical role in neural development in A3 knockout mice and under transient knockdown in mouse and human brain tissue [283]. Loss of A3 resulted in reduced neural progenitor cell abundance and loss of cortical thickness, while overexpression promoted neuronal proliferation. Mechanistically, Ou et al. [283] observed that A3 interacts with SMC1A (cohesin) during mitosis, regulating sister chromatid segregation. In the absence of A3, cell division was more frequently catastrophic and resulted in neuronal precursor death [283].

Pathobiologically, A3 has been linked to neurodegeneration, and specifically to ALS. A3 was found to preferentially bind *C9orf72*-derived hexanucleotide repeat (HR) RNAs, (GGGGCC)_n_ and (CCCCGG)_n_, that drive *C9orf72*-associated ALS [284,285]. These repeat sequences are pathologically expanded in the genomes of a subset of ALS patients, and are aberrantly translated into di-peptide repeat (DPR) molecules of GA (Gly-Ala), GR (Gly-Arg), PA (Pro-Ala), and PR (Pro-Arg) and GP (Gly-Pro), depending on sense and antisense reading frames, and the translational open reading frame [286]. Both the RNAs and the peptides are thought to be toxic and contribute to neurodegeneration, and A3 has been directly shown to interact with both [285,287]. A3 was also found to be dramatically mislocalized from its primarily-nuclear disposition to cytoplasmic diffuse and inclusion-body staining in the brains and spinal cords of ALS patients [284,287], while A1 and A2/B1 staining were unaffected [288]. The A3-containing inclusion bodies were found to be TDP-43-negative, Sequestosome-1 (p62)-positive, pathognomonic of *C9orf72* expansion-associated ALS [284,287,288,289]. Davidson et al. [287] propose that the association of A3 with the HR RNAs promotes their translation, exacerbating DPR-driven disease; however, the same group previously demonstrated that A3 knockdown resulted in an increase in GA accumulation and pathogenic RNA foci [289]. Instead, it appears that A3 reduces translation of HR RNAs, with A3 nuclear staining and GA staining intensities in patient tissues being inversely correlated [289]. Most recently, the effect of A3 nuclear depletion on DPR accumulation has been linked to increased DPR-driven DNA damage, providing a direct mechanistic link to the neuronal death that drives neurodegeneration [285].

Most of the pathology and mechanism of the role A3 plays in ALS has been characterized in the context of *C9orf72* expansion, but these publications have also included other ALS subtypes. Immunohistochemistry has shown A3 mislocalization to cytoplasm in brains and spinal cord of ALS patients without the genetic *C9orf72* expansion, but it is less severe, while control brain and spinal cord do not demonstrate A3 mislocalization at all [284,288]. Of interest, A3 also binds TDP-43, itself an aggregation-prone RBP with a PrLD that is implicated in ALS and that mislocalizes from nucleus to cytoplasm in neurodegenerative disease [290]. When TDP-43 was depleted, or its aggregation was induced, A3 mislocalized to the cytoplasm in response [291]. Ubiquilin 2 (*UBQLN2*), a protein regulator of autophagy, has been found to have genetic mutations associated with familial ALS [292], and A3 has demonstrated strong binding to wild-type Ubiquilin 2 that was lost for pathogenic ALS-associated *UBQLN2* mutations [293]. Gilpin et al. [293] found that interaction with Ubiquilin 2 stabilized A1 to maintain protein abundance, and presume that it would have a similar effect on A3, providing a connection to the other pathological A3 observations above. Tangentially, van Acker et al. [294] recently identified an interaction between A3 and neuroglobin, a neuroprotective protein that prevents ferroptosis in response to proteinopathy. During ferroptotic stress, neuroglobin interacts with A3, but the outcome of this interaction is unknown [294]. Altogether, these findings suggest a greater role for A3 in ALS pathogenesis than just HR and DPR regulation. However, despite both A1 and A2/B1 carrying missense mutations associated with ALS and neurodegenerative diseases [51,188], no single nucleotide variations in *HNRNPA3* in ALS patients (familial and spontaneous) have been detected in cohort screening studies [288,295,296].

## 6. hnRNP A/B Protein Structural Features

The majority of structural characterization has been carried out on regions of A1, with some few comparisons to A2/B1 (Table 2). No structural information for A0 nor A3 is available. Because of their relative structural stability, the globular RRM domains of both proteins have been characterized in detail, while researchers have only recently begun to examine the C-terminal LCD (PrLD) region in full [193,297], since the structure of this region is highly variable. Since this region is also less well-conserved relative to the RRMs, application of A1-derived structures to interpretation of A2/B1 and vice versa, or to the other hnRNP A/B family proteins, requires some caution.

### 6.1. Structures of Nucleic Acid-Unbound and -Bound RRMs in A1 and A2/B1 Proteins

**Figure 5:**
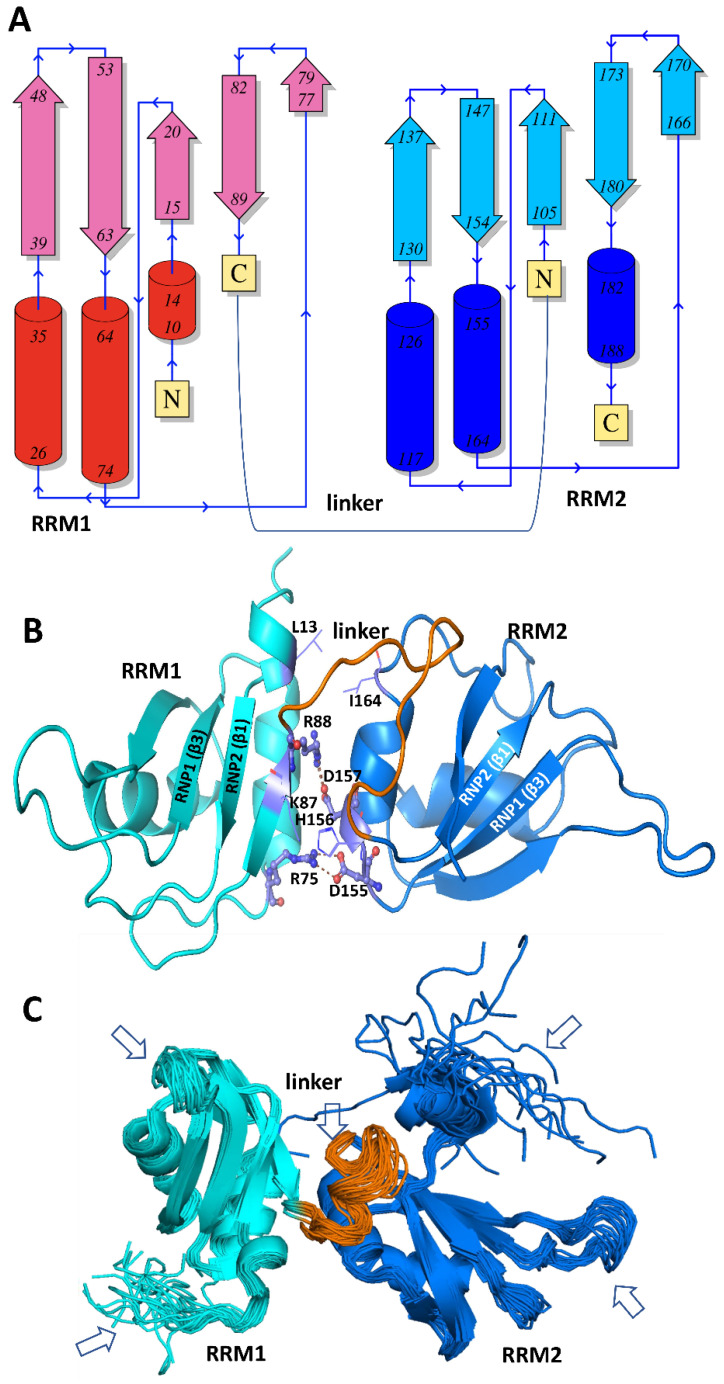
hnRNP A1 RRM structures. (**A**) The secondary structure topology diagram describes the β1α1β2β3α2β4 arrangements of both RRM1 and RRM2 in A1: four antiparallel β-sheets and two α helices as numbered in the figure. (**B**) A cartoon representation of the 3D structure of A1 RRMs (based on PDB: 1UP1), where RRM1 is shown in cyan and RRM2 in blue. The linker connecting the two RRMs is shown in orange. The key salt bridge interactions (shown as ball-and-stick representations) and hydrophobic contacts (residues shown in line format) at the interface of tandem RRMs are shown. The β-sheets encompassing RNP1 (β3) and RNP2 (β1) motifs in both the RRMs are marked in the structure. (**C**) A structural ensemble of 20 A1 RRM conformers (PDB code: 2LYV) are shown, and the flexible regions in the structures are marked with arrows.

The intradomain contacts of A1 RRMs in free state and their interactions with DNA and RNA at a molecular level have been characterized well. The A1 RRMs were originally characterized as “unwinding protein 1” or UP1, and span residues 1–196, with each RRM composed of ~90 residues (Figure 3). A1 RRM1 and A1 RRM2 share 35% amino acid identity and ~60% similarity [108], but are functionally non-equivalent [307]. Duplication, deletion, or swapping of A1 RRMs affected its alternative splicing properties [307], thus suggesting unique roles of the two RRMs in the functionality of A1. Thus far, three high-resolution crystal structures and one solution nuclear magnetic resonance (NMR) structure (encompassing 20 conformers) of free (or unbound) A1 RRMs have been reported (Table 1) [299,300,301,302]. These structures show that both RRM1 and RRM2 present the highly-conserved α/β fold of RRMs, four antiparallel β-sheets and two α helices packed as β1α1β2β3α2β4 (Figure 5A,B) [108]. The two RRMs are oriented in an anti-parallel manner and are interconnected by a ~17aa-long linker loop [308] that is positioned on the surface between the β-sheets of the RRMs. The RRMs mainly interact with each other through two salt-bridge interactions, ^RRM1^R75-D155^RRM2^ and ^RRM1^R88-D157^RRM2^ (Figure 5B). In addition, hydrophobic contacts mediated by ^RRM1^L13-I164^RRM2^ and ^RRM1^K87 -H156^RRM2^ also assist in stabilizing the RRM interface. An ensemble of 20 A1 RRM conformers determined by NMR in solution (PDB: 2LYV) [299] provided some insights into the dynamic behaviour of the structure. As expected, loop regions in the structures were mostly flexible, including the N-terminal loop, the C-terminal region, the long β2-β3 loops in each RRM, and the inter-RRM linker loop (Figure 5C). 

Each RRM further contains two conserved consensus RNP sub-motifs, dubbed RNP1 and RNP2, that together are crucial for RNA recognition [309]. In the A1 RRM1, the RNP1 is formed by an R55-G56-F57-F59-V60-T61 segment that forms the β3-strand, with the RNP2 sub-motif of K15-F17-G19-G20 is located on the β1 strand (Figure 4 and Figure 5B). Identical motifs form the RNP1 (R146-G147-F148-F150-V151-T152) and RNP2 (K106-F108-G110-G111) in RRM2 as well [302]. These residues for RNP1 and RNP2 are exposed on the surface of the RRMs and support RNA recognition. These residues comprising RNP1 and RNP2 are identical to A1 in human A2/B1 and A3 RRM1 and RRM2 regions (Figure 1), highlighting a strongly conserved motif and functionality, although there is a single amino acid variation in RNP1 the A0 RRM1 whose effect on RNA binding is unknown.

**Figure 6:**
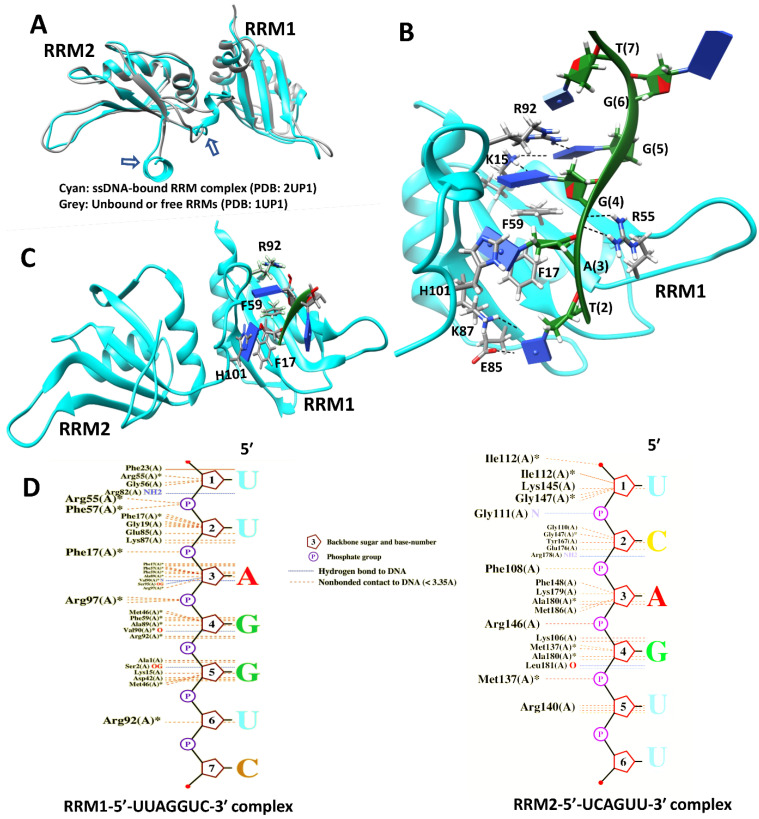
3D structures of DNA- and RNA-bound complexes of A1 RRMs, rotated forward 180° from Figure 5. (**A**) The structures of RRMs from the free-state (PDB: 1UP1, shown in gray) and the ssDNA-bound complex (PDB: 2UP1, shown in cyan) are overlapped to highlight the structural variations in the protein in response to DNA-binding. The linker loop and the C-terminal segment in RRMs became more ordered upon DNA-binding that led to the formation of short helices shown with arrows. (**B**) A closer view into the binding mode of 5′ end of ssDNA (shown as green ribbon and nucleotides shown as lines) against the surface of RRM1 (in cyan). The H-bond interactions and π-π between the protein residues and ssDNA are marked. (**C**) The 3D structure of A1 RRMs (cartoon representation in cyan) in complex with 5-AGU-3′ RNA (shown as green ribbon and nucleotides shown as lines). The key residues engaged in hydrophobic contact with the substrate are shown as stick representations. (**D**) 2D interaction diagrams showing the interactions between the RRM1-5′-UUAGGUC-3′ complex (left), and RRM2-5′-UCAGUU-3′ complex (right). The RNA molecules are shown as line drawing and key residues are noted. * denotes the residues that appear more than once in the plot.

Ding et al. [57] reported the first crystal structure of the hnRNP A1 RRMs bound with a 12-nucleotide long (d(TTAGGG)_2_) telomeric single-stranded DNA (ssDNA) (PDB: 2UP1), which provided extensive insights into nucleic acid recognition by A1 RRMs. The structure of this RRMs-ssDNA complex exhibited a symmetry-related dimer, in which two antiparallel ssDNA molecules interacted with two units of RRMs. In this state, the 5′half of the ssDNA interacted with the β-sheet surface of RRM1 in monomer 1, while the 3′ half of the ssDNA bound against the β-sheets of RRM2 in monomer 2. It was proposed that the dimer formation was triggered by the repeating nucleotides in the ssDNA. Structural alignment comparing this DNA-bound RRM complex to free RRMs (Figure 6A) reveals a ~15° shift in the relative positions of RRMs that highlights the compactness in the RRM structures when bound to the DNA. When compared to the free state, there were a few notable changes observed in the bound-complex: the β-sheets in the RRMs moved closer; the RRM-linker loop became more ordered; and a short helix was formed in the C-terminal of the RRMs (RRM2) in the bound-complex (Figure 6A).

A more detailed examination of A1 interaction with ssDNA can be achieved by focusing on the first unit of d(TTAGGG), the 5′ end of the ss-DNA used by Ding et al. [57], and its interaction with surface residues of RRM1 (Figure 6B). As will be shown for both DNA and RNA, the major points of nucleic acid contact in RRM1 are the A-G nucleotides with the residues F17 (RBP1) and F59 (RBP2), although the ssDNA sequence is also stabilized by hydrophobic and electrostatic interactions with several other residues. The first thymine (T(1)) was not resolved in the crystal structure, but T(2) made electrostatic interactions with the E85 and K87 residues located on the β4-sheet of RRM1. ssDNA recognition was mainly driven through the interactions of A(3)G(4)G(5) nucleotides and the RNP1/2 motifs (Figure 6B). A(3) was found to be sandwiched between the side-chain rings of F17 (from RNP2) and H101 from the linker loop, whereas G(4) interacted with the RNP1 residues F59 (stacking interactions) and R55 (hydrogen bonds; H-bonds). The G(5) nucleotide made H-bonds with K15 (an RNP2 motif) from β1-sheet of RRM1 and R92 from the linker loop. The last G nucleotide, G(6), was oriented away from the protein surface in an anti conformation. Similar interactions were seen between the 3′ half of ssDNA and RRM2, where the RNP1/2 residues again coordinated binding with the nucleotides [57]. Later crystal structures of A1 RRMs in complex with different modified telomeric ssDNA repeats exhibited very similar binding poses [303,304], and these structures confirmed that the H-bond interactions between the key residues of RRMs and the DNA played an important role in the structural stability. Together, the RRM-ssDNA complexes provided important molecular-level insights into ligand recognition processes by A1 RRMs.

Morgan et al. [305] generated the first experimental structure of a human A1 RRM-RNA complex (PDB: 4YOE), a high-quality crystal of an RRM-(5-AGU-3′) RNA complex, revealing the RNA bound in a similar manner to the ssDNAs with the RRMs (Figure 6C). The 3-nucleotide RNA interacted with the β-strands on RRM1 and the linker loop, while there was no contact between RRM2 and RNA. In this binding mode, the first A nucleobase made aromatic stacking interactions with H101 (from the linker) and F17 from RRM1, in addition to H-bonds with V90 and R88. In the case of the second G base, it involved in a cation-π contact with R92 and π-π interactions with F59 residue. The third base (U) adopted an anti conformation and was projected away from the RRM1 surface (Figure 6C) like G(6) in the DNA-bound structure. These interactions were very similar to those found in the RRM-DNA complexes, suggesting the specificity of RRM1 binding pocket towards the AG dinucleotide.

In another study, Beusch et al. [108] demonstrated RNA binding with solution structures of independent A1 RRM1 and A1 RRM2 using NMR experiments. In order to characterize the binding differences of RNA to each of the RRMs, they evaluated the binding of several short RNAs made up of 6–8 nucleotides to the isolated RRMs. They revealed that 5′-UUAGGUC-3′ RNA exhibited high affinity for RRM1, while 5′-UCAGUU-3′ showed specificity towards RRM2. The interactions of these complexes were described using the X-ray crystal structures (Table 2), and 2D interaction diagrams of these complexes are described in Figure 6D. The structure of the RRM1 complex (PDB: 5MPG) exhibited overall similarity with the previously-discussed RRM-DNA and RRM-RNA complexes, where the A(3)G(4) nucleotides were stacked against F17 and F59, respectively, along with electrostatic interactions with R88 and V90. The U(2) nucleotide was stabilized by hydrogen bond interactions with E85 and K87, while the flanking G(5) nucleotide engaged with D42 and R92 residues in RRM1, similar to what was observed with DNA binding [57]. In the case of the RRM2-5′-UCAGUU-3′ complex, the A(3)G(4) nucleotides were stabilized by F108 and F150 residues (RNP motifs in RRM2), which is similar to those interactions seen in the RRM1 complex. In addition, K179 and L181 made electrostatic interactions with A(3)G(4) dinucleotide. The C(2) nucleotide made several hydrophobic contacts (with G110, G147, E176) along with a H-bond with R178. Nevertheless, the three uracil nucleotides, U(1), U(6) and U(7) did not make any significant interactions with RRM2 and were highly disordered in the crystal structures. Thus, this work demonstrates that although both RRM1 and RRM2 in hnRNP A1 can bind RNA, its RRM1 is able to recognize longer RNA ligands than RRM2.

**Figure 7:**
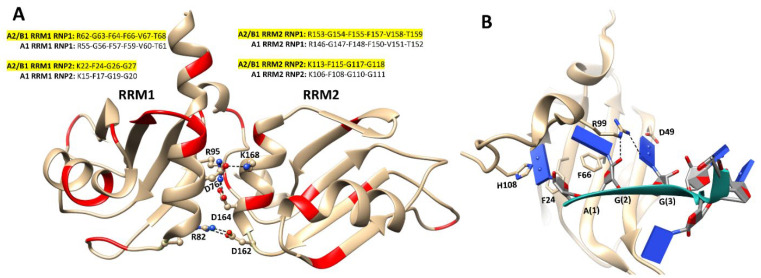
3D structures of hnRNP A2/B1 RRMs. (**A**) A cartoon representation of the 3D structure of A2/B1 RRMs (based on PDB: 5WWG), with residues that are different from the A1 RRMs coloured in red. The salt-bridge interactions at the interface of tandem RRMs are shown as ball and stick representations, and the consensus in the RNP1 and RNP2 motifs in A1 and A2/B1 RRMs (highlighted in yellow) are shown as text. (**B**) A closer view into the binding mode of a 10-mer RNA (shown as green ribbon with nucleotides shown as lines) against the surface of RRM1 (in yellow). The H-bond interactions and π-π between the protein residues (in stick representations) and RNA are marked.

Unlike the A1 RRMs, to date, there are only five crystal structures of A2/B1 RRMs in complex with 8-mer or 10-mer RNA substrates (Table 2) [306]. Most appear to use the B1 isoform, but only R12-K13-K14 of the isoform’s 12 amino acid insertion are resolved, and the best-resolved structure (PDB: 5HO4) does not include B1 residues at all. The RRMs of A2/B1 are each approximately 85 residues long each, connected by a 13-residue linker loop; in the A2 isoform, there is a 7-residue N-terminal tail preceding the RRMs, while this is elongated to 19 amino acids in the B2 isoform (Figure 3). Unsurprisingly, the structural fold of A2/B1 RRMs (in Figure 7A) resembles that of A1 protein (in Figure 5B). The interface of the RRMs in A2/B1 is also stabilized by three salt-bridge interactions, ^RRM1^D76-K168^RRM2^, ^RRM1^R95-D164^RRM2^, and ^RRM1^R82-D162^RRM2^ (using B1 numbered residues) comparable to those in A1. The sequence alignments based on the crystal structures of RRMs of A1 (PDB: 1UP1) and A2/B1 (PDB: 5WWG) show >80% identity between the two proteins’ RRM domains. The residues that are different in A2/B1 when compared to A1 are highlighted in red in Figure 7A, showing that the A2/B1 RRMs are largely identical to that of A1 protein, particularly within the β-sheets. Even the few variations seen in the β-strands of RRM2 of A2/B1 represent interchangeable substitutions (substitution to amino acids with similar properties): for example, I107 and A149 in A1 are changed to L114 and G156 in A2/B1, respectively (see Figure 1). Furthermore, the RNP1 and RNP2 in the RRMs of A2/B1 are 100% conserved when compared to those from A1 (see sequence comparisons of RNPs in Figure 7A).

The overall sequence-structure similarities between A2/B1 RRMs and A1 RRMs suggest that they could also exhibit similar RNA recognition processes at molecular-level. This was confirmed by the crystal structures of RNA-bound A2/B1 RRM complexes reported by Wu et al. [306]. For example, the binding mode of an 8-mer RNA (5′AGGACUGC-3′) against the A2/B1 RRM1 (Figure 7B) was very similar to the binding poses of RNA-A1-RRM1 and DNA-A1 RRM1 and interactions. In the RNA-A2/B1 RRM binding mode, the A(1) of the 8-mer RNA made π-π interactions with F24 and H108; G(2) was stacked between the sidechains of F66 and R99; and G(3) engaged in H-bond interactions with D49 and R99 residues. The other nucleotides in the 8-mer RNA did not make any significant interactions with the RRMs. In the same work, the authors characterized the interactions of a slightly longer 10-mer RNA that had both AGG and UAG motifs with the A2/B1 RRMs. Interestingly, this crystal structure presented a dimer of A2/B1 RRM interacting with 2 anti-parallel 10-mer RNAs, in which the AGG motif bound on the surface of RRM1 of monomer 1 and the UAG motif was recognized by the RNPs in RRM2 [306]. This binding mode was very similar to that presented by the ssDNA-RRM complex in A1 [57,303,304].

In summary, the known structures of A1 and A2/B1 RRMs indicate that these globular domains are able to specifically recognize the ‘AGG’ nucleotide motif. The ‘AGG’ motif preferentially binds the ‘nucleotide pocket’ formed on the surface by the β-strands of RRM1 and the linker loop. Although the consensus RNPs in RRM2 are also able to recognize and bind ‘AGG’ (and ‘UAG’) motifs, they are only able to recognize much shorter substrates. When the substrate is longer and has repeating nucleotides, then the RRMs form dimer interactions, where a pair of anti-parallel ligand binds with RRM1 in monomer 1 (through its 5′ end) and RRM2 in monomer 2 (via its 3′ end). There is currently no structure that demonstrates the binding of a single ssDNA or RNA interacting with the tandem RRMs in monomer state. Thus, the ligand likely mainly engages with the RRM1 surface, whereas RRM2, despite its own ability to interact with the ligand, is probably only playing an auxiliary role in ligand recognition. More experiments are required to test this hypothesis.

### 6.2. Structures of LCD Fibrils of A1 and A2/B1 Proteins

The C-terminal glycine-rich LCD of hnRNP A/B proteins forms fibrils due to cellular stress, and more readily does so when mutated in neurodegenerative diseases. Therefore, revealing the molecular processes associated with the functional and disease-related fibrils in these proteins is important. To address this, the structures of LCD fibrils of human A1 (PDB: 7BX7) [193] and A2/B1 (PDB: 6WQK) [177] proteins were recently solved through cryogenic electron microscopy (cryo-EM; Table 2). These structures provide insights into molecular interactions that facilitate fibril formation in these proteins.

**Figure 8:**
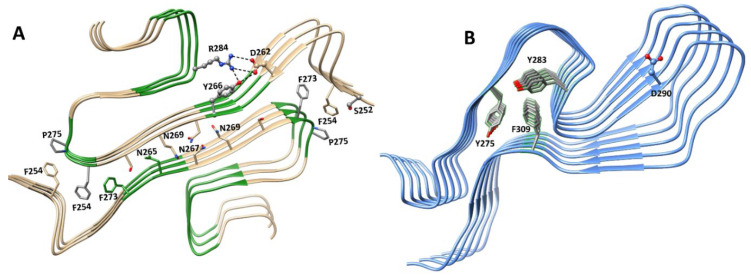
3D Structures of A1 and A2/B1 LCD fibrils. (**A**) A cartoon representation of a 3D fibril structure (PDB: 7BX7) formed by multimers of the A1 LCD residues 251–295, with the key residues engaging in different electrostatic and van der Waals interactions are shown in stick representations. (**B**) A cartoon representation of a 3D fibril structure (PDB: 6WQK) formed by multimers of the A2 LCD residues 263–319 shown in blue. The key aromatic stacking interactions rendered by Y275, Y283, and F309 are shown as sticks. The D290 residue, which is mutated to a valine residue (D290V) in ALS, is rendered in ball-and-stick mode for context.

Fibrils were resolved encompassing residues 251–295 in A1 (Figure 8A). This segment of the protein is rich in glycines, which results in coils that are present on either side of the fibril, forming β strands composed of residues 260–273. The C-terminal coils are oriented in parallel (Figure 8A) to the protofilaments that promote a three-way interaction between Y266, D262 (through electrostatic contacts) and R284 (via a cation-π contact), which helps in increasing the stability of the fibrils. The interface of fibrils exhibits a steric zipper arrangement, which is a known phenomenon in fibrils [297,310]. In this zipper mode, the side chains of N265, N267, Q269, and S271 engage in van der Waals interactions to form a stable and complementary formation. At the ends of this zipper, the hydrophobic contacts of the F263-F273 pair and the F254-P275 pair enclose the core zipper and protect it from the water molecules that enhance the stability of the fibrils (Figure 8A). Since most of the residues in the M9-NLS motif (Figure 3) are involved in fibril formation in the cryo-EM structure, Sun et al. suggest that interaction of TNPO1 with the NLS—which facilitates the return of A1 to the nucleus—could prevent cytoplasmic protein aggregation in A1 [193]. Apart from this fibril structure (PDB: 7BX7), the experimental 3D structures of different short LCD segments have also been reported in the PDB (Table 2).

The structure of LCD fibrils in hnRNP A2 protein (PDB: 6WQK) spanning residues 263–319 was also recently resolved through cryo-EM technology [297]. The structure (Figure 8B) represents a steric zipper fibril form that includes four short β-sheets formed by residues G274-N277, Y288-D290, P303-S306, and S312-N314. Aromatic stacking interactions formed between three residues, Y275, Y283, and F309, are located in the center of the fibrils. Aromatic Y and F residues that promote this stacking [311] are apparent at regular intervals in the linear structures of all the hnRNP A/B proteins, visualized best in Figure 4. Similar to the A1 LCD fibrils, the M9 NLS motif in A2 (Figure 3) was also found to be involved in the fibril formation. Lu et al. [297] estimated the solvation energy of this fibril structure of A2 to be −19.5 kcal mol^−1^ (per chain), which is much smaller than that seen in the disease-linked irreversible amyloids (~ −34 kcal mol^−1^ per chain). Thus, the wild-type LCD fibrils reported in the cryo-EM structure are less stable and more likely reversible. In comparison, fibrils of the D290V A2 mutant associated with ALS [188] exhibit greater stabilization energy [297], thus providing insights into D290V mutant-driven protein aggregation in neurodegenerative diseases.

In summary, experimental 3D structures of the N-terminal globular RRMs and the C-terminal fibril forms in hnRNP A1 and A2/B1 have provided extensive insights about the molecular interactions underlying the physiological and pathophysiological processes in the proteins. At the same time, there is increasing evidence that RRMs could have an allosteric impact on the C-terminal protein aggregation in these proteins [179,312], but structures of the full protein for any of the hnRNP A/B family have yet to be resolved. Therefore, understanding the structure-dynamic relations of the complete A1 or A2/B1 proteins will be required to understand these hidden molecular-level processes and develop potent inhibitors for various neurological disorders.

## 7. Conclusions

**Figure 9:**
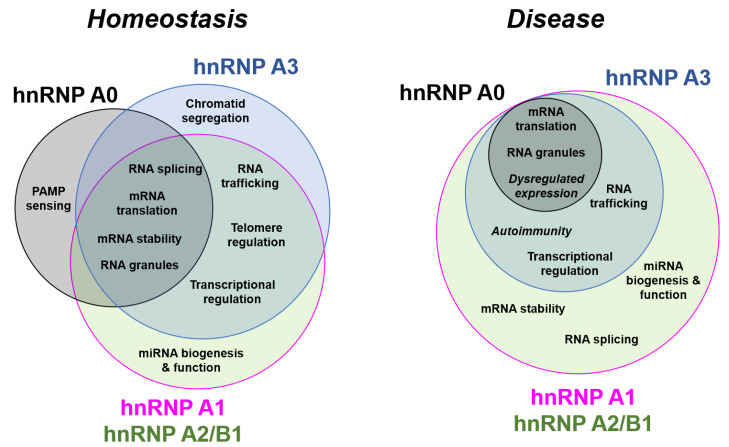
Known shared and unique functions of hnRNP A/B family proteins in homeostasis and in disease. Future research will likely alter this understanding.

This review has provided a comprehensive examination of the A/B family proteins and their structures, as well as their functions in homeostasis and disease (Figure 9). Even from such a high-level perspective, it is clear that there is much left to be studied about these critical proteins. A/B proteins are all closely related, share significant sequence homology, and have highly conserved domain architecture and structures, yet they carry out separate functions using distinct mechanisms. Even when comparing the relatively well-characterized A1 and A2/B1 proteins, literature suggests that they differ in the ways they regulate RNA biology, but it is often not clear whether these are due to unique properties of each protein, or whether the protein has yet to be examined for a given function. Similarly, in many instances, researchers have identified sites of post-translational modification on A1, and simply assumed that similar modifications will occur with similar stimulus at a homologous site on A2/B1. However, there is remarkably little evidence that the extensive post-translational modifications found in A1 are echoed A2/B1, and what evidence exists suggests that A2/B1 has reduced levels of modification. What determines this difference and the difference in A/B protein responses to stimulus, is worth exploration. This connects to the need for an understanding of what drives their alternative and/or pathogenic cytoplasmic functions in the context of tissues and organisms, rather than cell culture, in order to design therapeutic interventions for a number of diseases. Aside from A0, these proteins also have tightly-regulated and evolutionarily-conserved splice variants, indicating that different isoforms perform distinct, essential functions in the context of the cell, tissue, or organism. Despite this clear signature, there is limited literature exploring the functional differences between isoforms of a given protein, and virtually no discussion of 5′ and 3′ UTR variants of the genes’ mRNAs, which are likely to include distinct regulatory features. Such exploration is complicated by A1 and A2/B1, which regulate their own splicing, such that modulating isoform abundance is likely to feed back in complicated ways. Recent forays into structural analyses have finally started to provide information on the C-terminal glycine-rich LCDs, but there is a need for complete structures of these proteins to generate a holistic understanding of how protein:protein interaction, aggregation, and RNA binding activities of both N- and C-terminal domains regulate homeostatic, stress response, and pathogenic functions of A/B family proteins. In the context of neurodegenerative disease, we see a pressing need for development of therapeutic molecules that modulate specific activities (namely, aggregation and nuclear-cytoplasmic translocation) of these proteins, which necessitates advancements in our understanding of their structures and protein:protein interactions.

## Figures and Tables

**Table 1:** Basic characteristics of hnRNP A/B family proteins.

Common Protein Name	Gene Name ^a^ (Ensembl ID)	Chromosomal Location	Isoforms ^b^ (in Order of Abundance)	Protein Length (aa)	References
**hnRNP A0**	*HNRNPA0*	Ch5q31.2, rev	n/a	**305**	[33]
	(ENSG00000177733)				
**hnRNP A1**	*HNRNPA1*	Ch12q13.13, for	**hnRNP A1**	**320**	[34,35]
	(ENSG00000135486)		**hnRNP A1-B**	**372**	
**hnRNP A2/B1**	*HNRNPA2B1*	Ch7p15.2, rev	**hnRNP A2**	**341**	[18,36,49,50]
			**hnRNP B1**	**353**	
	(ENSG00000122566)		hnRNP A2b	301	
			hnRNP B1b	313	
			hnRNP A2*	253	
**hnRNP A3**	*HNRNPA3*	Ch2q31.2, for	**hnRNP A3 var 1**	**378**	[31,39,40]
			**hnRNP A3 var 2**	**356**	
	(ENSG00000170144)		hnRNP A3 var 3	331	
			hnRNP A3 var 4	309	

^a^ All information is derived from human hnRNPs. ^b^ Note that isoforms refer only to protein isoforms, and do not encompass variant 5′ and 3′ UTRs in the mRNAs. Highlighted isoforms refer to primary isoforms, and bolded isoforms refer to relatively abundant isoforms.

**Table 2:** Three dimensional (3D) experimental structures of hnRNP A1 and A2/B1 reported in the protein data bank (PDB).

PDB Code	Structure Description	Technique (Resolution in Å)	Refs	Year
hnRNP A1				
7BX7	A1 LCD (residues 251–295)	Cryo-EM (2.8)	[193]	2020
6BXX	A1 LCD (residues 243–248)	X-ray diffraction (1.1)	[298]	2018
5ZGL	A1 LCD (residues 234–240)	X-ray diffraction (0.95)	[185]	2019
6J60	A1 LCD (residues 209–217)	Electron crystallography (0.96)	[185]	2019
2LYV	A1 RRMs	Solution NMR	[299]	2013
1L3K	A1 RRMs	X-ray diffraction (1.1)	[300]	2002
1HA1	A1 RRMs	X-ray diffraction (1.75)	[301]	1997
1UP1	A1 RRMs	X-ray diffraction (1.9)	[302]	1997
1U1L/1U1K/ 1U1R/1U1Q/ 1U1P/1U1O/ 1U1N/1U1M	A1 RRMs complexed with modified telomeric DNA repeats	X-ray diffraction (1.8–2.1)	[303]	2004
1PO6/ 1PGZ	A1 RRMs complexed with DNA	X-ray diffraction (2.1/2.6)	[304]	2003
2UP1	A1 RRM-single-stranded telomeric DNA complex	X-ray diffraction	[57]	1999
6DCL	A1 RRMs-bound to pri-miRNA-18a	X-ray diffraction (2.5)	[144]	2018
5MPG	A1 RRM1 bound to 5′-UUAGGUC-3′	Solution NMR	[108]	2017
5MPL	A1 RRM2 bound to 5′-UCAGUU-3′	Solution NMR	[108]	2017
4YOE	A1 RRMs bound to 5’-AGU-3’ RNA	X-ray diffraction (1.92)	[305]	2015
hnRNP A2/B1				
6WQK	A2 LCD (residues 263–319)	Cryo-EM (3.1)	[297]	2020
6WPQ	D290V mutant A2 LCD (residues 286–291)	X-ray diffraction (1.10)	[297]	2020
5WWE/ 5WWF/5WWG	A2/B1 RRMs in complex with 10-mer RNA	X-ray diffraction (2.03–2.15)	[306]	2018
5HO4	A2/B1 RRMs in complex with 10-mer RNA	X-ray diffraction (1.85)	[306]	2018
5EN1	A2/B1 RRMs in complex with 8-mer RNA	X-ray diffraction (2.58)	[306]	2018

## Data Availability

Publicly available datasets were analyzed in this study. This data can be found here: https://www.ncbi.nlm.nih.gov/gene/3178/ortholog/ (accessed on 20 April 2021), https://www.ncbi.nlm.nih.gov/gene/3181/ortholog/ (accessed on 20 April 2021), https://www.ncbi.nlm.nih.gov/gene/220988/ortholog/ (accessed on 20 April 2021), https://www.ncbi.nlm.nih.gov/gene/10949/ortholog/ (accessed on 20 April 2021). Other data is referenced throughout the article.
